# Synthesis of Dense 1,2,3-Triazole Oligomers Consisting Preferentially of 1,5-Disubstituted Units via Ruthenium(II)-Catalyzed Azide–Alkyne Cycloaddition

**DOI:** 10.3390/polym15092199

**Published:** 2023-05-05

**Authors:** Ryoichi Taguchi, Masaki Nakahata, Yuri Kamon, Akihito Hashidzume

**Affiliations:** 1 Department of Macromolecular Science, Graduate School of Science, Osaka University, 1-1 Machikaneyama-cho, Toyonaka 560-0043, Osaka, Japan; 2 Administrative Department, Graduate School of Science, Osaka University, 1-1 Machikaneyama-cho, Toyonaka 560-0043, Osaka, Japan

**Keywords:** click chemistry, ruthenium(II)-catalyzed azide–alkyne cycloaddition, 1,5-disubstituted 1,2,3-triazole, dense 1,2,3-triazole polymer

## Abstract

Ruthenium(II)-catalyzed azide–alkyne cycloaddition (RuAAC) polymerization of *t*-butyl 4-azido-5-hexynoate (tBuAH), i.e., a heterobifunctional monomer carrying azide and alkyne moieties, was investigated in this study. RuAAC of the monofunctional precursors of tBuAH yielded a dimer possessing a 1,5-disubstituted 1,2,3-triazole moiety. ^1^H NMR data showed that the dimer was a mixture of diastereomers. Polymerization of tBuAH using ruthenium(II) (Ru(II)) catalysts produced oligomers of *M*_w_ ≈ (2.7–3.6) × 10^3^ consisting of 1,5-disubstituted 1,2,3-triazole units (1,5-units) as well as 1,4-disubstituted 1,2,3-triazole units (1,4-units). The fractions of 1,5-unit (*f*_1,5_) were roughly estimated to be ca. 0.8 by comparison of signals of the methine and triazole protons in ^1^H NMR spectra, indicating that RuAAC proceeded preferentially and thermal Huisgen cycloaddition (HC) somehow took place during the polymerization. The oligomer samples obtained were also characterized by solubility test, size exclusion chromatography (SEC), ultraviolet-visible (UV-Vis) absorption spectroscopy, and thermogravimetric analysis (TGA). The UV-Vis and TGA data indicated that the oligomer samples contained a substantial amount of Ru(II) catalysts. To the best of our knowledge, this is the first report on dense 1,2,3-triazole oligomers consisting of 1,5-units linked via a carbon atom.

## 1. Introduction

Biological systems utilize polymers, i.e., proteins, nucleic acids, and polysaccharides, to maintain their living activities, e.g., genetic transcription, control of chemical reactions, and energy storage. Bio-inspired synthetic polymers, consisting of functional building blocks linked through a variety of bonds, have been designed and synthesized [1,2,3]. Appropriate design of the polymer structure, e.g., the building blocks and the linkers between the building blocks, may allow one to synthesize polymers that exhibit various functions inspired by biological systems. One of the promising residues that mimic a peptide bond is a 1,2,3-triazole ring [4,5,6,7,8,9,10]. The formation of the 1,2,3-triazole ring by 1,3-dipolar cycloaddition of azide and alkyne moieties, i.e., Huisgen cycloaddition (HC), is promising and reliable for the synthesis of polypeptide mimics based on its high reaction selectivity and efficiency [11]. While thermal HC yields mixtures of 1,4- and 1,5-disubstituted 1,2,3-triazoles, HC catalyzed by Cu(I) compounds forms regioselectively 1,4-disubstituted 1,2,3-triazoles (copper(I)-catalyzed azide–alkyne cycloaddition (CuAAC)) [12,13,14]. In contrast, Ru(II) compounds possessing bulky ligands, e.g., cyclopentadienyl (Cp) and pentamethylcyclopentadienyl (Cp*), are also known to catalyze HC reaction, giving preferably 1,5-disubstituted 1,2,3-triazoles (ruthenium(II)-catalyzed azide–alkyne cycloaddition (RuAAC)) [15,16,17,18]. RuAAC has also been used for the synthesis of functional polymers [19,20,21].

We have been focusing on dense 1,2,3-triazole polymers obtained from 3-azido-1-propyne (AP) derivatives [22,23,24,25]. Recently, we reported the synthesis of 4-azido-5-hexynoic acid (AH) derivatives possessing azide and alkyne moieties as monomers for CuAAC polymerization [26,27,28]. We also polymerized the AH derivatives by CuAAC to obtain polymers possessing 1,4-disubstituted 1,2,3-triazole moieties linked via a methine (C1) linker, which can be regarded as analogs of poly(glutamate) because a 1,2,3-triazole moiety has structural features similar to those of a peptide bond [4,5,6,7,8,9,10]. The polymers formed from AH derivatives by CuAAC consisted of 1,4-disubstituted 1,2,3-triazole units (1,4-units). AH derivatives also underwent thermal HC polymerization without any catalyst to form polymers consisting of 1,4-units and 1,5-disubstituted 1,2,3-triazole units (1,5-units). It is noteworthy that the fractions of 1,4- and 1,5-units (*f*_1,4_ and *f*_1,5_, respectively) depended on the polarity of the solvent used for thermal HC polymerization [26]. Since the polymers of AH derivatives, of which the sequence of 1,4- and 1,5-units is controlled, should take a variety of conformations, it is worth studying the RuAAC of AH derivatives.

**Scheme 1:**
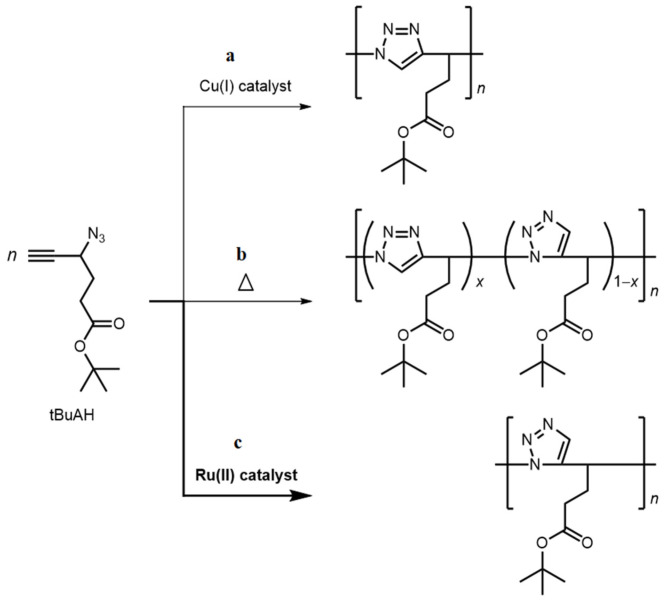
Polymerizations of tBuAH via CuAAC with a Cu(I) catalyst (**a**), thermal HC without any catalyst (**b**), and RuAAC with a Ru(II) catalyst (**c**).

In this work, we report the applicability of Ru(II) catalysts in the polymerization of *t*-butyl 4-azido-5-hexynoate (tBuAH) as a monomer (Scheme 1). We first investigate RuAAC of model compounds possessing either azide or alkyne moiety to obtain a dimer linked via a 1,5-unit. Then, we study RuAAC polymerization of tBuAH using two Ru(II) catalysts and characterize the samples obtained. The properties of the samples are compared with those of a poly(tBuAH) sample consisting of 1,4-units obtained by CuAAC.

## 2. Materials and Methods

### 2.1. Materials

Dichloromethane, *N*,*N*-dimethylformamide (DMF), dimethyl sulfoxide (DMSO), ethyl acetate, hexane, methanol, tetrahydrofuran (THF), lithium bromide (LiBr), and chloro(1,5-cyclooctadiene)(*η*^5^-pentamethylcyclopentadienyl)ruthenium(II) (Cp*RuCl(COD)) were purchased from FUJIFILM Wako Pure Chemical Corp. (Osaka, Japan). A solution of tetrabutylammonium fluoride (TBAF) in THF (1 M) was purchased from Tokyo Chemical Industry Co., Ltd. (Tokyo, Japan). Copper(I) bromide (CuBr), sodium chloride (NaCl), sodium sulfate (Na_2_SO_4_), and tetrasodium ethylenediaminetetraacetate (EDTA·4Na) were purchased from Nacalai Tesque, Inc. (Kyoto, Japan). Pentamethylcyclopentadienylbis(triphenylphosphine)ruthenium(II) chloride (Cp*RuCl(PPh_3_)_2_) was purchased from Sigma–Aldrich (St. Louis, MO, USA). Merck precoated TLC plates (silica gel 60 F254) were used for thin layer chromatography (TLC) analysis in this work. Silica gel 60 (Nacalai Tesque, spherical, neutrality) was used for column chromatography to purify the products. THF and DMF were dried with flame-dried molecular sieves 4A. Other reagents were used without further purification. tBuAH and *t*-butyl 6-(*t*-butyldimethylsilyl)-4-hydroxy-5-hexynoate (**1**) were prepared according to the procedure reported previously [26].

### 2.2. Measurements

^1^H NMR spectra were recorded on a JEOL (Tokyo, Japan) JNM ECS400 or ECA500 spectrometer at 25 °C using chloroform-*d* (CDCl_3_) or dimethyl sulfoxide-*d*_6_ (DMSO-*d*_6_) as a solvent. Chemical shifts were determined using the signal due to tetramethylsilane (TMS) (0 ppm) as an internal standard. For the rough estimation of the fractions of 1,4-unit (*f*_1,4_) in the RuAAC and CuAAC products, the area intensity of the signal due to 1,4-disubstituted 1,2,3-triazole CH (ca. 8.2–8.6 ppm) was compared with that due to the methine CH (ca. 6.6–5.6 ppm). The fractions of 1,5-unit (*f*_1,5_) was then estimated assuming *f*_1,4_ *+ f*_1,5_ = 1. Electrospray ionization mass spectra (ESI-MS) were collected in a positive ion mode using a Thermo Fisher Scientific (Waltham, MA, USA) LTQ Orbitrap-XL, controlled by the XCARIBUR 2.1 software package. Methanol (high-performance liquid chromatography (HPLC) grade) was employed as a solvent. The condition of ionization was set as follows; ion spray voltage at 3.5 kV, ion spray temperature at 100 °C, and ion transfer tube temperature at 275 °C. Internal calibration of ESI-MS was carried out using the monoisotopic peaks of sodium-adducted ion of diethylphthalate, protonated ion of di-2-ethylhexylphthalate, and sodium-adducted ion of di-2-ethylhexyl-phthalate (*m*/*z* = 314.1410, 391.2843, and 413.2662, respectively). Size exclusion chromatography (SEC) measurements were performed at 40 °C on a JASCO (Tokyo, Japan) ChromNAV system with a TSKgel SuperAW-H guard column and two TOSOH TSK SuperAWM-H columns using DMSO containing LiBr (1.05 g L^−1^) as an eluent. The flow rate was set to 1.0 mL min^−1^. Poly(ethylene glycol) (PEG) standards (Scientific Polymer Products, Inc. (Ontario, NY, USA)) were utilized for the calibration of molecular weights. A sample solution (10 g L^−1^, 100 μL) was injected into the SEC instrument after filtration with an Advantec (Tokyo, Japan) DISMIC-13JP PTFE 0.50 μm. Ultraviolet-visible (UV-Vis) absorption spectra were collected on a MERCK (Darmstadt, Germany) Spectroquant Prove 600 spectrophotometer using a 1.0 cm path-length quartz cuvette at 25 °C. DMF was used as a solvent. All the data were subtracted with background (i.e., the solvent only). Thermogravimetric analysis (TGA) data were recorded on a Hitachi Hightech (Tokyo, Japan) NEXTA STA300 instrument using an aluminum sample pan as the temperature was increased from 30 °C to 600 °C at a heating rate of 20 °C min^−1^. The weights of samples were within the range of 1.2–1.6 mg.

### 2.3. Synthesis of t-butyl 4-hydroxy-5-hexynoate (**2**)

A solution of TBAF in THF (1 M, 4.7 mL, 4.7 mmol) was added dropwise to a solution containing compound **1** (680 mg, 2.3 mmol) in dry THF (25 mL) at 0 °C. The reaction solution was stirred at 0 °C for 3 h. After stirring, the reaction solution was poured into water (50 mL). The product was extracted with ethyl acetate (3 × 30 mL), and the organic layers were combined. The combined organic phase was washed with saturated NaCl (50 mL). The organic phase was dried with Na_2_SO_4_, and the Na_2_SO_4_ was then removed by filtration. Volatile fractions were removed under reduced pressure. The product was purified by column chromatography using a mixed solvent of hexane and ethyl acetate (20/1–1/1, *v*/*v*). After evaporation of volatile fractions under reduced pressure, compound **2** was obtained as a colorless oil (285 mg, 1.55 mmol, 67.7%). ^1^H NMR (400 MHz, 298 K, CDCl_3_): *δ* 4.48 (s, 1H, CH), 2.57–2.40 (m, 2H, CH_2_), 2.61 (s, 1H, OH), 2.48 (d, *J* = 2.0 Hz, 1H, CH≡C), 2.07–1.94 (m, 2H, CH_2_), 1.45 (s, 9H, *t*-C_4_H_9_).

### 2.4. Model Reaction of RuAAC Using Compounds **1** and **2**

Compounds **1** (65 mg, 0.20 mmol) and **2** (37 mg, 0.20 mmol) were dissolved in THF (0.3 mL) at room temperature under a nitrogen atmosphere. Then, a solution of Cp*RuCl(PPh_3_)_2_ (35 mg, 0.044 mmol) in THF (1.7 mL) was added to the reaction mixture. The reaction solution was stirred at 60 °C for 4 h. Volatile fractions were removed under reduced pressure. The product was purified by column chromatography using a mixed solvent of hexane and ethyl acetate (5/1–2/1, *v*/*v*). After the removal of volatile fractions under reduced pressure, compound **3** was obtained as a brown oil (34 mg, 66 μmol, 33%). ^1^H NMR (400 MHz, CDCl_3_) *δ* 7.61 (s, 1/2H, triazole CH), 7.60 (s, 1/2H, triazole CH), 5.73–5.60 (m, 1H, CH), 5.18–4.98 (m, 1H, CH), 3.51 (d, *J* = 4.7 Hz, 1/2H, OH), 3.45 (d, *J* = 5.3 Hz, 1/2H, OH), 2.65–2.10 (m, 8H, CH_2_), 1.46–1.38 (s, 18H, *t*-C_4_H_9_), 0.92 (s, 9H, *t*-C_4_H_9_), and 0.11 (s, 6H, CH_3_).

### 2.5. Model Reaction of CuAAC Using Compounds **1** and **2**

Compounds **1** (93 mg, 0.29 mmol) and **2** (71 mg, 0.39 mmol) were dissolved in a mixed solvent of *t*-butanol and water (2/1, *v*/*v*) (1.5 mL) under a nitrogen atmosphere. CuSO_4_·5H_2_O (10.2 mg, 0.04 mmol) and sodium ascorbate (22.1 mg, 0.11 mmol) were added to the solution under a nitrogen atmosphere. The reaction solution was stirred at 60 °C for 19 h. After the addition of water (15 mL) to the reaction solution, the mixture was extracted with ethyl acetate (3 × 15 mL) and washed with saturated NaCl (20 mL). After the organic layer was dried with Na_2_SO_4_, volatile fractions were removed under reduced pressure to obtain a yellow oil (129 mg). Recrystallization from a mixed solvent of hexane and diethyl ether (1/1, *v*/*v*) at −20 °C yielded compound **4** as a colorless solid (38.7 mg, 76 μmol, 27%). ^1^H NMR (400 MHz, CDCl_3_) *δ* 7.73 (s, 1/2H, triazole CH), 7.72 (s, 1/2H, triazole CH), 5.56–5.52 (m, 1H, CH), 5.00–4.94 (m, 1H, CH), 3.13 (d, *J* = 4.8 Hz, 1/2H, OH), 3.08 (d, *J* = 4.8 Hz, 1/2H, OH), 2.48–2.08 (m, 8H, CH_2_), 1.46–1.45 (d, *J* = 0.8 Hz, 9H, *t*-C_4_H_9_), 1.44 (d, *J* = 0.8 Hz, 9H, *t*-C_4_H_9_), 0.95 (s, 9H, *t*-C_4_H_9_), and 0.14 (s, 6H, CH_3_).

### 2.6. RuAAC Polymerization of tBuAH

A typical example of RuAAC polymerization of tBuAH is described below.

tBuAH (1.00 g, 4.79 mmol) was dissolved in DMF (5.0 mL) at room temperature under a nitrogen atmosphere. Then, Cp*RuCl(PPh_3_)_2_ (421 mg, 0.529 mmol) was added to the reaction solution. The reaction solution was stirred at 50 °C for 8 h using a Biotage Initiator+ microwave reactor (Uppsala, Sweden) (2.45 GHz, the high adsorption level). The reaction solution was diluted with ethyl acetate (5.0 mL) and added dropwise to hexane (50 mL). The suspension was centrifuged at 4000 rpm for 10 min to recover the product as a precipitate. After drying under reduced pressure, the product was obtained as a brown solid (367 mg, 36.7%).

### 2.7. Density Functional Theory (DFT) and Time-Dependent DFT (TDDFT) Calculations

The initial molecular structures were constructed using Chem3D software (version 21.0.0.28) installed on a Windows 10 computer. DFT and TDDFT calculations were carried out using the Gaussian 09 program [29]. In calculations of the total energies and UV-Vis spectra for diastereomers, for compounds **3** and **4**, i.e., **3RR**, **3RS**, **4RR,** and **4RS** (see Figure S1 in Supplementary Materials), DFT and TDDFT with B3LYP functional were used, respectively, and 6-31G(d,p) basis sets were applied for the hydrogen, carbon, and nitrogen atoms. All the geometries of **3RR**, **3RS**, **4RR,** and **4RS** were fully optimized.

## 3. Results and Discussion

### 3.1. Model Reaction of RuAAC

**Figure 1:**
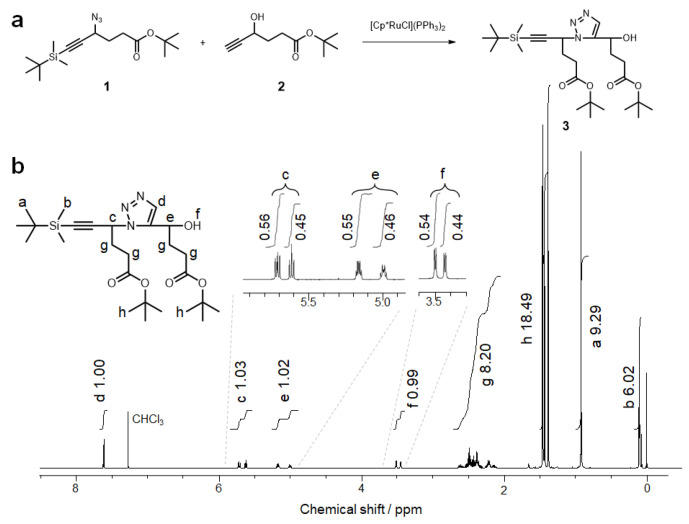
(**a**) Scheme for preparation of a model compound (**3**) from hexynoate derivatives (**1** and **2**) via RuAAC. (**b**) ^1^H NMR spectrum for **3** (CDCl_3_). The inset indicates magnified views of signals c, e, and f.

RuAAC of tBuAH derivatives was investigated using model compounds to confirm the formation of a 1,5-disubstituted 1,2,3-triazole. Figure 1a shows a synthetic scheme for compound **3** from compounds **1** and **2** using Cp*RuCl(PPh_3_)_2_ as a catalyst. Compound **1** is a precursor of tBuAH possessing a TBDMS-protected alkyne moiety, and compound **2** is a precursor carrying a hydroxy group that can be converted to an azide. The model reaction was carried out using 22 mol% of Cp*RuCl(PPh_3_)_2_ in THF at 60 °C for 4 h. Compounds **1** and **2** were consumed quantitatively, but the yield of compound **3** was as low as 33%, presumably because of the formation of byproducts, e.g., an isomer of compound **3** possessing a 1,4-disubstituted 1,2,3-triazole moiety and a dimer of compound **1** (Figure S2) [15]. Compound **3** was characterized by ^1^H and ^13^C NMR, and MS, as can be seen in Figure 1b and Figures S3 and S4. The ^1^H NMR spectrum of compound **3** shows a signal due to the 1,5-disubstituted 1,2,3-triazole at 7.6 ppm (c.f., a signal due to the 1,4-disubstituted 1,2,3-triazole at 7.7 ppm (Figure S5) [26]). The NMR and MS data confirmed that RuAAC was successful in forming compound **3**. It should be noted here that the signals c, e, and f in Figure 1b were split into two signals, and the integral ratios for signals at the lower and higher magnetic fields were approximately 0.55:0.45. Since compound **3** has two chiral carbon atoms, these signals are ascribable to the diastereomers.

### 3.2. RuAAC Polymerization

RuAAC polymerization of tBuAH was then investigated. The conditions and results of RuAAC polymerizations of tBuAH are summarized in Table 1. Two common Ru(II) catalysts for RuAAC were employed: Cp*RuCl(PPh_3_)_2_ and Cp*RuCl(COD) [16]. Since our preliminary study indicated that microwave irradiation enhanced the regioselectivity, all the reactions in this study were carried out using a Biotage Initiator+ microwave reactor at 50 °C for 8 h in DMF [17]. The concentration of tBuAH was fixed at 1.0 mol L^−1^. The concentration of Ru(II) catalysts was varied, i.e., 5, 10, and 20 mol%. For reference, CuAAC polymerization of tBuAH was also carried out using CuBr (Run 7 in Table 1) [26]. (RuAAC polymerizations were also conducted at 40 and 60 °C, but the results were practically the same.) Each reaction mixture was poured into a 10-fold volume of hexane, and the obtained precipitate was collected by centrifugation, washed with hexane, and then dried under reduced pressure. ^1^H NMR spectra for polymerization mixtures confirmed the quantitative conversion of the monomer (data not shown). In the cases of Cp*RuCl(PPh_3_)_2_ and Cp*RuCl(COD), the yield shows a tendency to increase with the catalyst concentration. The yields (88% and 67%) for 20 mol% Cp*RuCl(PPh_3_)_2_ and Cp*RuCl(COD) were higher than that of the CuAAC polymerization (56%). The *M*_w_ values determined by SEC (Figure S6) were (2.7–3.6) × 10^3^, which was lower than that for the CuAAC polymerization (5.9 × 10^3^). (It may not be able to directly compare these *M*_w_ values for samples obtained by RuAAC and CuAAC because the polymer chains take different conformations.) These observations indicate that CuAAC polymerization proceeded more efficiently than RuAAC polymerization. The conditions of RuAAC polymerization should be further optimized to obtain higher molecular-weight polymers in the near future.

### 3.3. Characterization of the Oligomer Samples Obtained

**Figure 2:**
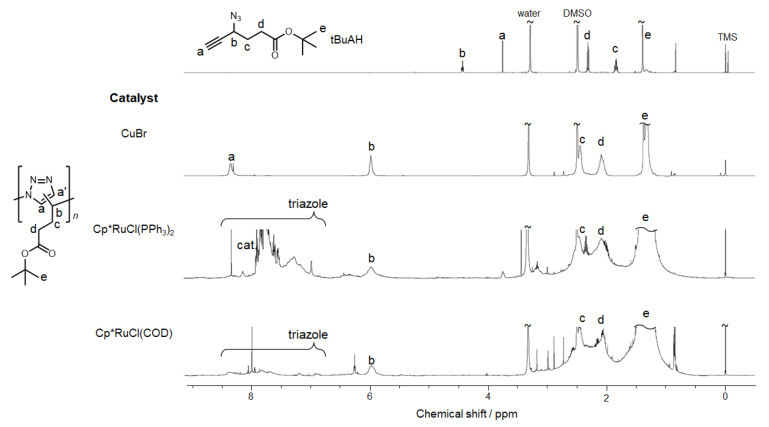
^1^H NMR spectra for tBuAH, poly(tBuAH) via CuAAC polymerization, and samples obtained via RuAAC polymerization (DMSO-*d*_6_).

The oligomer samples were characterized by ^1^H NMR to examine the regioselectivity. Figure 2 shows ^1^H NMR spectra of the oligomer samples together with the spectrum of tBuAH. All the ^1^H NMR spectra for the oligomer samples indicate no signals due to the alkyne proton, indicative of quantitative conversion of tBuAH. In all the spectra, signal e and signals c and d are attributed to the tBu group and the methylene protons in the tBuAH unit. The ^1^H NMR spectrum of tBuAH contains the signal due to the methine proton at ca. 4.5 ppm, whereas the spectra of the oligomer samples exhibit the methine signals at ca. 6 ppm. The signals in the aromatic region (8.5–6.5 ppm) observed in the spectra of the products are assignable to the 1,2,3-triazole proton. Our previous study demonstrated that thermal HC polymerization of tBuAH produced polymers consisting of both 1,4- and 1,5-units, while CuAAC polymerization yielded polymers consisting of 1,4-units [26]. The oligomer samples obtained in Runs 1–6 should preferably consist of 1,5-units. Unfortunately, since it was not possible to remove the Ru(II) catalyst completely from the samples during the purification procedure (i.e., column chromatography using silica or alumina, washing with aqueous solutions of chelating agents, or treatment with activated carbon), ^1^H NMR spectra contained signals due to the catalyst in the aromatic region. We roughly estimated the fractions of 1,4-unit (*f*_1,4_) of the products because the signal due to the proton in 1,4-unit was observed separately (Figure S7 and Table S1), and calculated the fractions of 1,5-unit (*f*_1,5_) using the value of *f*_1,4_, as listed in Table 1. The *f*_1,5_ values were ca. 0.8, indicating that RuAAC proceeded preferentially to produce the 1,5-units, and thermal HC somehow took place to form the 1,4-units. It should be noted here that *f*_1,5_ was practically constant at ca. 0.8, independent of the Ru(II) catalyst concentration. These observations imply that it is difficult to obtain polymers of *f*_1,5_ ≈ 1 presumably because of the steric effect of a longer sequence of 1,5-units.

The solubilities of the oligomer sample of Run 2 in Table 1 were tested using conventional solvents (Table 2). This table also contains the results of the solubility test for a polymer sample consisting of 1,4-units obtained by CuAAC polymerization. It is noteworthy that the oligomer sample obtained by RuAAC polymerization was soluble in a wider variety of solvents other than hexane and water compared to the sample obtained by CuAAC polymerization. This may be partly because the *M*_w_ was rather low (3.7 × 10^3^), and the polymer conformation was more compact for the sample obtained by RuAAC.

**Figure 3:**
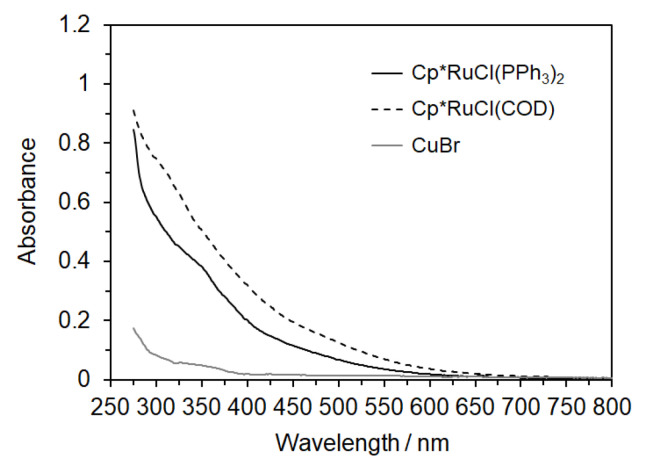
UV-Vis spectra for poly(tBuAH) samples obtained by RuAAC (Runs 2 (solid line, black) and 5 (dashed line, black) in Table 1) and CuAAC (Run 7 (solid line, gray) in Table 1) polymerizations (0.1 g L^−1^ in DMF).

**Figure 4:**
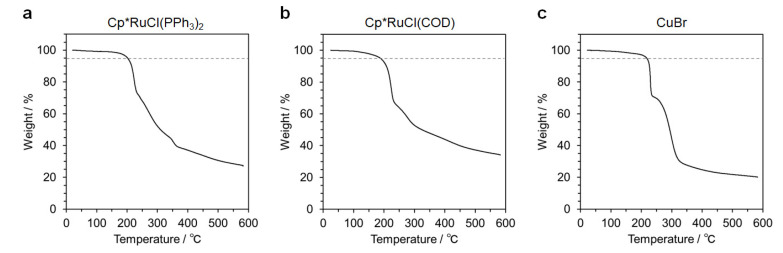
Thermogravimetric traces for poly(tBuAH) samples obtained by RuAAC (Runs 2 (**a**) and 5 (**b**) in Table 1) and CuAAC (Run 7 (**c**) in Table 1) polymerizations.

The oligomer samples of Runs 2 and 5 in Table 1 were also characterized by UV-Vis absorption spectrometry and TGA. (Cp*RuCl(PPh_3_)_2_ and Cp*RuCl(COD) were utilized in Runs 2 and 5, respectively.) Figure 3 shows UV-Vis spectra for 0.1 g L^−1^ solutions of the samples (Runs 2 and 5) in DMF. For comparison, this figure also contains the spectrum for the sample of Run 7, in which CuBr was used. The spectra for Runs 2 and 5 show stronger absorption over the UV to Vis range. Figure S7 shows the optimized structures and simulated UV-Vis spectra for the diastereomers of model compounds **3** and **4** (**3RR**, **3RS**, **4RR**, and **4RS**) obtained by DFT and TDDFT calculations, respectively. The simulated spectra are almost the same, which show absorption bands in the UV region (*λ* < 300 nm). It is thus likely that the absorption observed for Runs 2 and 5 is indicative of the remaining Ru(II) catalysts in the samples. Figure 4 shows TGA traces for the samples of Runs 2, 5, and 7. All the samples show significant weight loss by ca. 30 *w*/*w*% around 200 °C, which is ascribable to the dissociation of *t*-butyl ester in the side chains. The 5 *w*/*w*% weight loss temperature, which is an index of thermal stability, was 202, 188, and 218 °C for the samples of Runs 2, 5, and 7, respectively. These data indicate that the samples obtained by RuAAC were less thermally-stable than the sample obtained by CuAAC. The amounts of the residue remaining after heating up to 600 °C were 27 and 34 *w*/*w*% for the samples of Runs 2 and 5, respectively, which were larger than that for the sample of Run 7 (20 *w*/*w*%). These observations indicate that the oligomer samples adsorbed strongly Ru(II) ions.

## 4. Conclusions

RuAAC polymerizations of tBuAH were investigated using Cp*RuCl(PPh_3_)_2_ and Cp*RuCl(COD) as Ru(II) catalysts to obtain polymers consisting preferentially of 1,5-units. The polymerizations of tBuAH in the presence of the Ru(II) catalysts produced oligomer samples of *f*_1,5_ ≈ 0.8. These observations indicate that RuAAC proceeded preferably, and thermal HC somehow took place during the polymerization. The oligomer samples were soluble in a wider variety of solvents other than hexane and water. The UV-Vis spectra and TGA data indicated that the oligomer samples contained a significant amount of the Ru(II) catalysts, indicating that the oligomer samples of *f*_1,5_ ≈ 0.8 also adsorbed strongly metal ions. The combination of our previous study [26] and this work may allow one to synthesize poly(tBuAH) with controlled fractions of 1,4- and 1,5-units in the range of 0 ≤ *f*_1,5_ ≤ 0.8. To the best of our knowledge, this work is the first report on dense 1,2,3-triazole oligomers consisting of 1,5-units linked via a carbon atom (C1). Regio-, stereo-, and sequence-controlled 1,2,3-triazole oligomers and polymers linked via C1 linkers [28], which can be analogs for oligopeptides and proteins, will be designed and synthesized as bio-inspired synthetic oligomers and polymers in our future work.

## Figures and Tables

**Table 1:** Conditions and characterization of resultant polymers for RuAAC and CuAAC polymerizations.

Run	Temperature, Time	Catalyst	Catalyst/mol%	Yield/%	*M*_w_ ^1^/10^3^	*M*_w_/*M*_n_	*f*_1,5_ ^2^
1	50 °C, 8 h	Cp*RuCl(PPh_3_)_2_	5	55	2.9	1.4	0.81
2	50 °C, 8 h	Cp*RuCl(PPh_3_)_2_	10	37	3.6	1.9	0.85
3	50 °C, 8 h	Cp*RuCl(PPh_3_)_2_	20	88	2.8	1.6	0.85
4	50 °C, 8 h	Cp*RuCl(COD)	5	27	3.2	1.4	0.86
5	50 °C, 8 h	Cp*RuCl(COD)	10	42	2.7	1.3	0.82
6	50 °C, 8 h	Cp*RuCl(COD)	20	61	3.1	1.4	0.79
7	50 °C, 8 h	CuBr	10	56	5.9	1.7	0

^1^ Determined by SEC measurements at 40 °C using DMSO containing 1.05 g L^−1^ LiBr as eluent and PEG as standard. ^2^ The molar fraction of 1,5-unit determined by ^1^H NMR.

**Table 2:** Results of solubility test for poly(tBuAH) samples obtained by RuAAC (Run 2 in Table 1) and CuAAC polymerizations.

Solvent	Solubility ^1^	
	RuAAC ^2^	CuAAC ^3^
DMSO	++	++
DMF	++	++
THF	++	++
chloroform	++	++
dichloromethane	++	++
acetone	++	++
ethyl acetate	++	+
acetonitrile	++	+
methanol	++	−
toluene	++	−
diethyl ether	++	−
hexane	−	−
water	−	−

^1^ “++”, “+”, and “−” denote soluble at ≥10 g L^−1^, soluble at <10 g L^−1^, and insoluble, respectively. ^2^ Samples from Run 2 (Table 1) were used. ^3^ Results from Yamasaki et al. [26].

## Data Availability

The data presented in this study are available on request from the corresponding author.

## Supplementary Materials

The following supporting information can be downloaded at: https://www.mdpi.com/article/10.3390/polym15092199/s1, Figure S7: DFT calculations and simulated UV-Vis spectra for diastereomers of compounds **3** and **4**; Figure S2: ESI-MS for compound **3** (crude); Figure S3: ^13^C NMR spectrum for compound **3** (CDCl_3_); Figure S4: ESI-MS for compound **3**; Figure S5: Scheme for preparation of a model compound possessing 1,4-disubstituted 1,2,3-triazole (**4**) from **1** and **2** via CuAAC and ^1^H NMR spectrum for **4**; Figure S6: SEC traces for samples obtained via RuAAC and CuAAC polymerizations; Figure S7: Magnified ^1^H NMR spectra to determine *f*_1,4_ ratio; Table S1: Integral values to determine *f*_1,4_ and *f*_1,5_.
