# Supplementary material for: Synthesis of Dense 1,2,3-Triazole Oligomers Consisting Preferentially of 1,5-Disubstituted Units via Ruthenium(II)-Catalyzed Azide–Alkyne Cycloaddition
Source: Polymers (Basel). 2023 May 5;15(9):2199. doi: 10.3390/polym15092199 (PMC10180885; doi:10.3390/polym15092199)
Supplement: Supplementary file 1 [file polymers-15-02199-s001.zip › polymers-2347624-supplementary.pdf]

polymers

MDPI

Supporting Materials

Synthesis of Dense 1,2,3-Triazole Oligomers Consisting Preferentially of 1,5-Disubstituted Units via Ruthenium(II)-Catalyzed Azide–Alkyne Cycloaddition

Ryoichi Taguchi [1], Masaki Nakahata [1], Yuri Kamon [2] and Akihito Hashidzume [1,*]

1. Department of Macromolecular Science, Graduate School of Science, Osaka University, 1-1 Machikaneyama-cho, Toyonaka, Osaka 560-0043, Japan; taguchir21@chem.sci.osaka-u.ac.jp (R.T.); nakahata@chem.sci.osaka-u.ac.jp (M.N.)
2. Administrative Department, Graduate School of Science, Osaka University, 1-1 Machikaneyama-cho, Toyonaka, Osaka 560-0043, Japan; kamon@chem.sci.osaka-u.ac.jp

* Correspondence: hashidzume@chem.sci.osaka-u.ac.jp; Tel.: +81-6-6850-8174

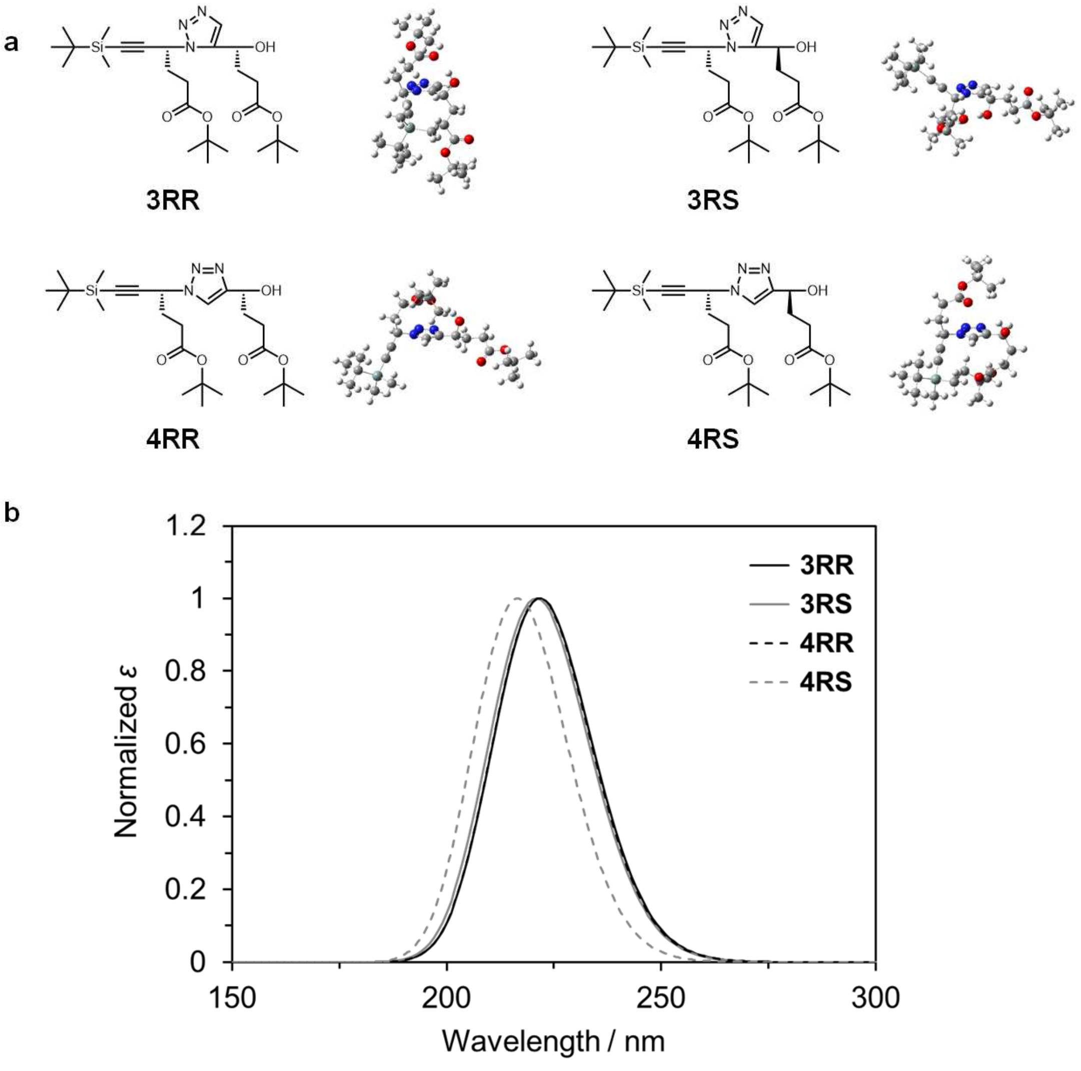

Figure S1. (a) Chemical structures and DFT optimized structures calculated for compounds **3RR**, **3RS**, **4RR** and **4RS**. (b) Simulated UV-Vis spectra for compounds **3RR**, **3RS**, **4RR** and **4RS**.

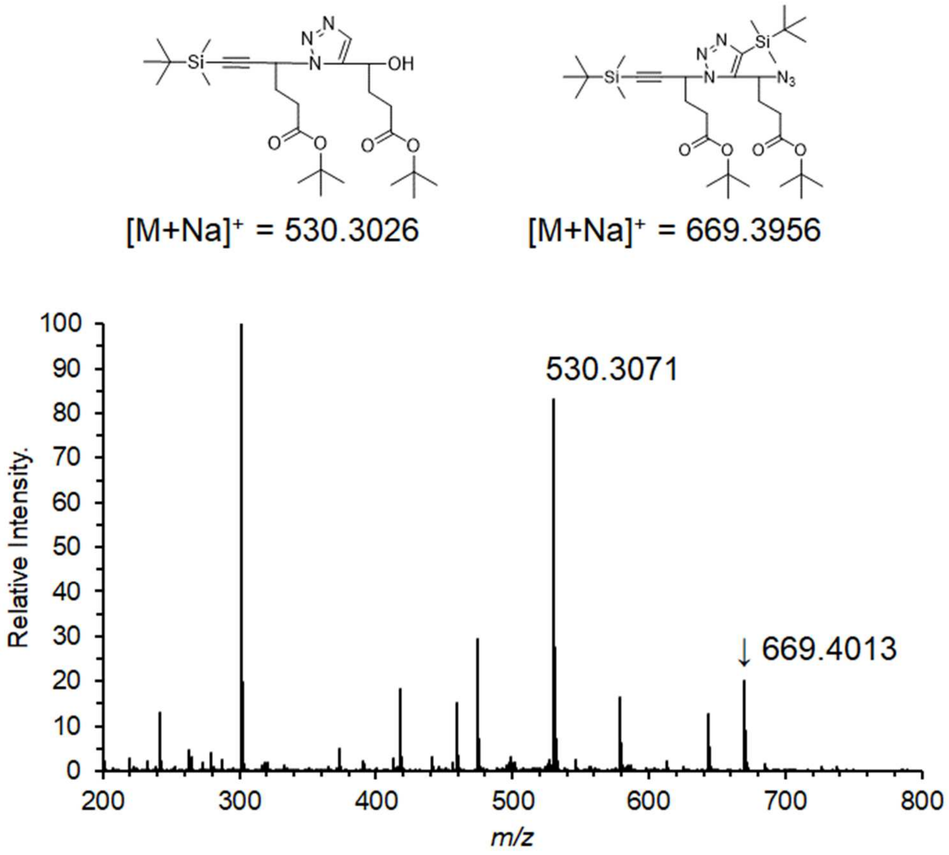

Figure S2. ESI-MS for compound **3** (crude).

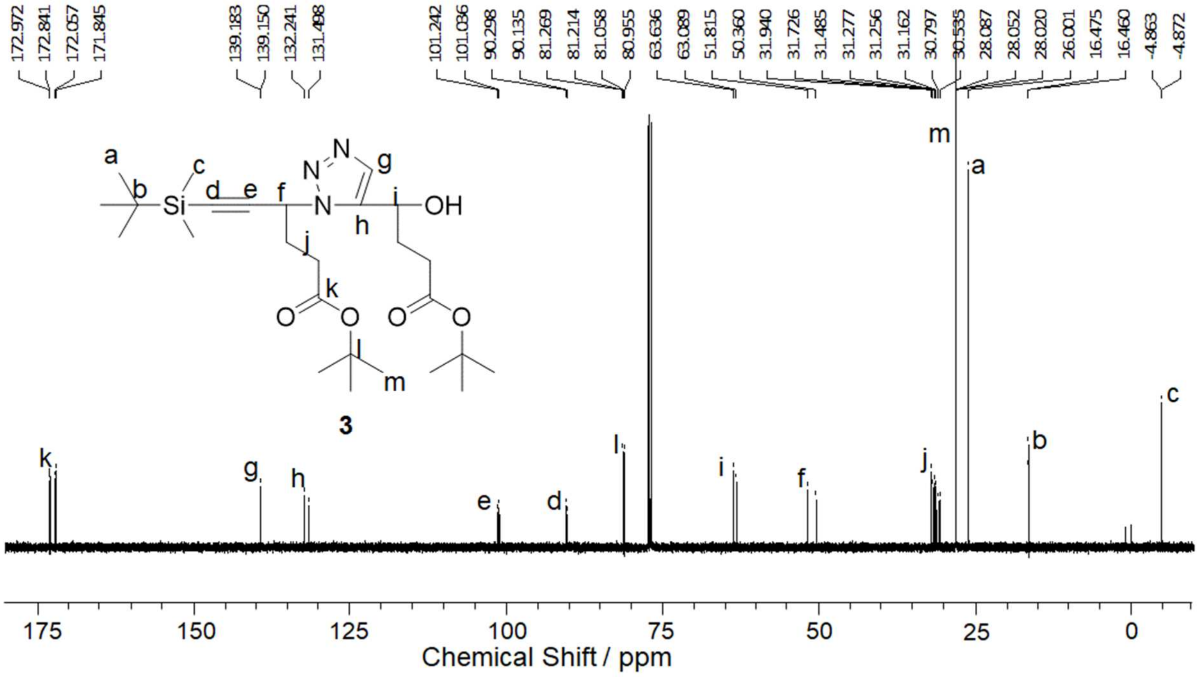

Figure S3. ^{13}C NMR spectrum for compound **3** ($CDCl_3$).

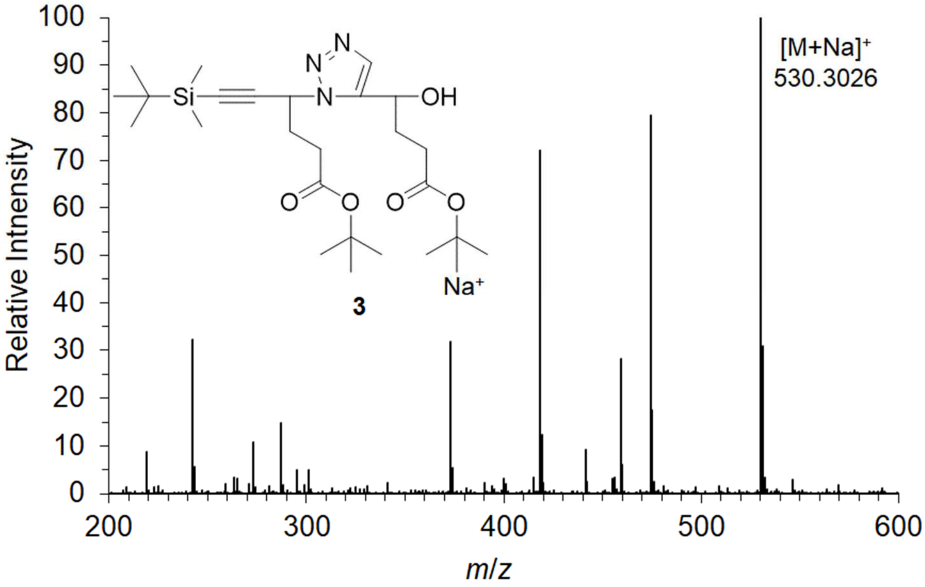

Figure S4. ESI-MS for compound **3**.

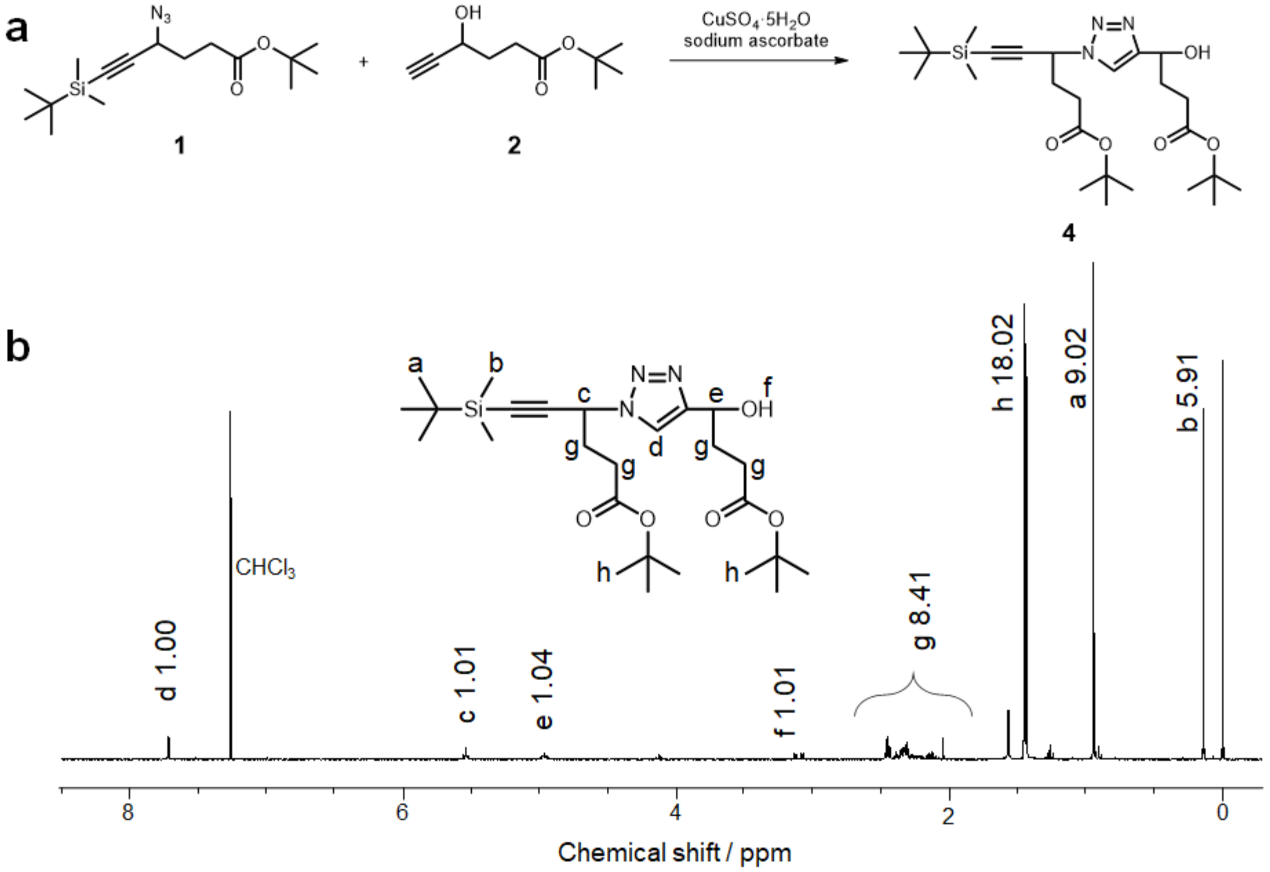

Figure S5. (a) Scheme for preparation of a model compound possessing 1,4-disubstituted 1,2,3-triazole (**4**) from **1** and **2** via CuAAC. (b) ^{1}H NMR spectrum for **4** ($CDCl_3$).

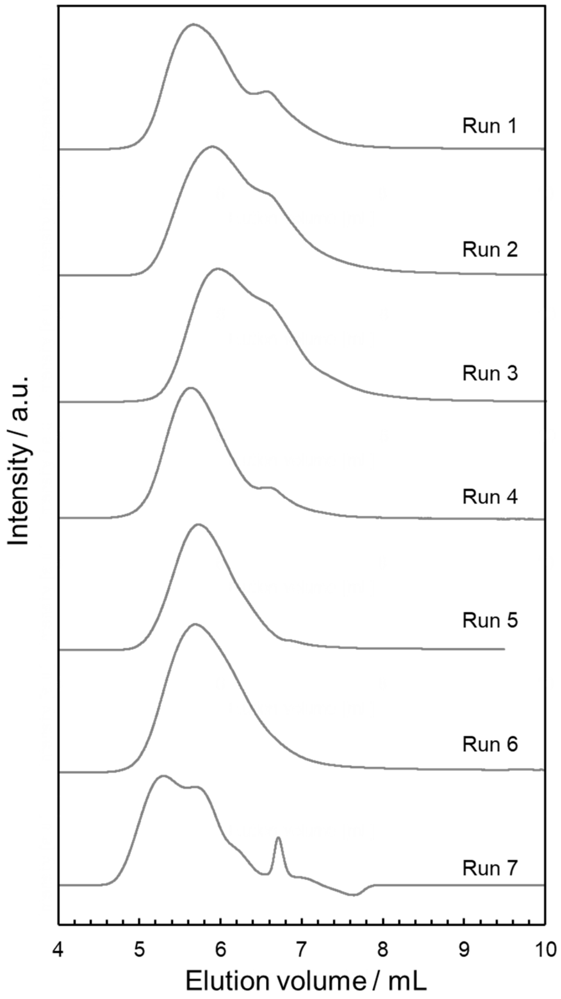

Figure S6. Size exclusion chromatography (SEC) traces for samples obtained via RuAAC and CuAAC polymerizations (Eluent: DMSO containing LiBr (1.05 g L^{-1}), flow rate: 1.0 mL min^{-1}). Run numbers correspond to those in Table 1.

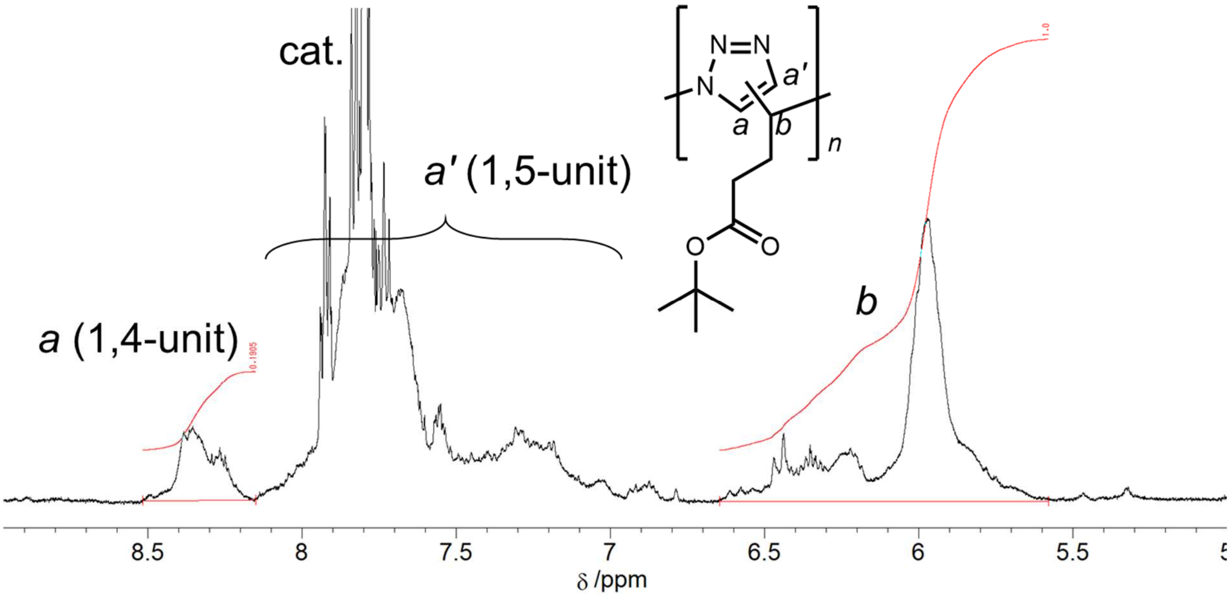

Figure S7. Magnified ^{1}H NMR spectrum for Run1 in Table 1 with integral values for methine protons (right) and 1,4-triazole protons (left) (DMSO-d_6).

Table S1. Relative integral values to estimate $f_{1,4}$ and $f_{1,5}$ for Runs in Table 1.

Run	Integral value (*a*)	Integral value (*b*)	$f_{1,4}$	$f_{1,5}$
1	0.1905	1	0.19	0.81
2	0.1487	1	0.15	0.85
3	0.1538	1	0.15	0.85
4	0.1436	1	0.14	0.86
5	0.1780	1	0.18	0.82
6	0.2132	1	0.21	0.79
7	1.0034	1	1	0
